# Innovative Hurdle Strategies for *Listeria* Control on Food-Contact Surfaces: A Peroxyacetic Acid–Steam Approach

**DOI:** 10.3390/foods13162481

**Published:** 2024-08-07

**Authors:** Zi Hua, Mei-Jun Zhu

**Affiliations:** School of Food Science, Washington State University, Pullman, WA 99164, USA; zi.hua@wsu.edu

**Keywords:** *Listeria* control, food-contact surface, cross-contamination, hurdle strategies, peroxyacetic acid, thermal

## Abstract

The persistence of *Listeria monocytogenes* biofilms on equipment surfaces poses a significant risk of cross-contamination, necessitating effective surface decontamination strategies. This study assessed the effectiveness of hurdle treatments combining peroxyacetic acid (PAA) and saturated steam against 7-day-old *L. innocua* (a non-pathogenic surrogate for *L. monocytogenes*) biofilms on stainless steel (SS), polyester (PET), and rubber surfaces. Results demonstrated >6 log_10_ CFU/coupon *L. innocua* reductions on SS and PET surfaces after PAA (40 ppm, 1 min) followed by steam treatment (100 °C, 6 s). On rubber surfaces, PAA (80 ppm, 1 min) followed by steam treatment (100 °C, 6 s) resulted in ~5 log_10_ CFU/coupon *L. innocua* reduction. The presence of apple juice soil reduced the efficacy of hurdle treatments, with PAA (40 ppm, 1 min) and steam exposure (6 s) resulting in 5.6, 5.8, and 4.2 log_10_ CFU/coupon reductions of *L. innocua* on SS, PET, and rubber, respectively. The efficacy of this antimicrobial combination was further reduced by surface defects, especially in the presence of organic matter. Nevertheless, the treatment still achieved >5 log_10_ CFU/coupon reductions of *L. innocua* on worn SS and PET soiled with apple juice and ~4.5 log_10_ CFU/coupon reduction on worn, soiled rubber surfaces. These findings highlight that PAA treatments followed by a brief steam exposure are effective strategies for controlling *Listeria* on food-contact surfaces.

## 1. Introduction

Contamination of food-contact surfaces by *Listeria monocytogenes* is of great concern in the fresh produce industry. *L. monocytogenes* has been frequently isolated from direct food-contact surfaces and production environments in fresh produce facilities [1,2,3,4,5,6]. The unsanitary packinghouse environments and processing lines have been recognized as the major reasons for multi-state listeriosis outbreaks associated with cantaloupes [7] and caramel apples [8]. Also, surfaces of contaminated equipment in direct contact with fresh produce have been implicated in the recalls of various foods, including fresh-cut products [9], stone fruits [10], citrus products [11,12], and pre-packaged salads [13]. This information highlights the challenge of *L. monocytogenes* contamination on food-contact surfaces and underscores the insufficiency of current cleaning and sanitation practices in eliminating *L. monocytogenes*. Thus, there is an urgent need to explore effective strategies for decontaminating food-contact surfaces.

To reduce *L. monocytogenes* contamination on food-contact surfaces, chemical sanitizers such as chlorine dioxide, hypochlorite, quaternary ammonium compound, and peroxyacetic acid (PAA) are commonly utilized before or after each production shift [14]. Although chlorine is the most extensively used sanitizer for surface decontamination, its reaction with organic matter could produce carcinogenic by-products [15], causing additional food safety concerns. PAA could quickly decompose into acetic acid and water without generating harmful by-products; thus, it is safe for surface disinfection [16]. Also, PAA has emerged as one of the most effective sanitizers for disinfecting *L. monocytogenes* biofilms from food-contact surfaces [17,18,19,20]. When treating the 7-day-old biofilms on stainless steel (SS), the 1 min use of PAA at 160 ppm caused a 4.3 log_10_ CFU/coupon reduction of *L. monocytogenes* [18]. However, the disinfection efficacy of PAA was adversely impacted by surface conditions, particularly organic matter [17,18] and surface defects [21]. For example, a 1 min exposure to 160 ppm PAA resulted in a 3.8 log_10_ CFU/coupon reduction of *L. monocytogenes* in disinfecting the 7-day-old biofilms on worn SS surfaces, with efficacy further reduced in the presence of organic matter [21]. On the other hand, steam is a traditional thermal approach used infood processing and equipment decontamination, generating no chemical residue and being environmentally friendly. Due to the large amount of heat, steam treatments have exhibited potential to inactivate *Listeria* biofilms. For instance, a short-time steam exposure (5–6 s) caused a rapid kill of *Listeria* biofilms on SS [22,23,24]. A 6 s exposure to saturated steam led to a 3.1 log_10_ CFU/coupon reduction of *Listeria* on SS [24]. However, extending steam exposure beyond 6 s induced *Listeria* survival tail on various surfaces [24,25]. These data indicated that saturated steam or PAA treatment, when used alone, may be inadequate to eliminate *Listeria* biofilms. Therefore, there remains a critical need for alternative and effective sanitization strategies to eliminate *Listeria* contamination on food-contact surfaces.

Previous studies have investigated hurdle interventions combining sanitizer treatments with moist heat [26,27] or steam exposure [22,23] for eradicating *L. monocytogenes* contamination on SS surfaces. For example, a 30 s exposure to chlorine at 50 ppm followed by a 30 s heating at 65 °C resulted in a 4.8 log_10_ CFU/cm^2^ reduction of *L. monocytogenes* adhered to SS, starting from a pre-treatment level of ~5.0 log_10_ CFU/cm^2^ [26]. A combination of short-duration steam treatments and lactic acid [23], chlorine, benzalkonium chloride, or hydrogen peroxide [22] has effectively enhanced the efficacy of a single steam or sanitizer treatment in disinfecting the biofilms of *L. monocytogenes* on new SS. Hydrogen peroxide treatment (2%, 30 s) followed by a 10 s contact with saturated steam resulted in ~4 log_10_ CFU/coupon reduction of *L. monocytogenes* in biofilms on SS from an initial level of ~6 log_10_ CFU/coupon, in comparison to <2 log_10_ CFU/coupon reduction achieved by steam or hydrogen peroxide treatment alone [22]. Findings from those studies demonstrated the potential of steam–sanitizer hurdle treatment to eradicate *Listeria* biofilms from food-contact surfaces. However, it is unknown for the efficacy of saturated steam in combination with PAA against *L. monocytogenes* biofilms. Additionally, most steam–sanitizer hurdle inactivation studies have been conducted on SS surfaces, and there is no information about the effectiveness of this PAA–steam hurdle treatment in decontaminating biofilms from non-SS surfaces, including polyester (PET, materials used for brush filaments in brush-bed system) and rubber (materials for conveyor system), which are heat-tolerant materials [28,29]. Moreover, the surfaces of SS, PET, and rubber can become worn with increased usage and may also be soiled with organic matter if cleaning is insufficient. Yet, limited information is available regarding the influence of surface defects and food soils on the sanitization efficacy against *Listeria* biofilms, which warrants further research.

Therefore, the objectives of this study were to (1) examine the efficacy of PAA treatments combined with quick saturated steam exposure for decontaminating 7-day-old *L. innocua* biofilms on surfaces including SS, PET, and rubber; (2) assess how the treatment sequence of saturated steam and PAA affects the effectiveness of hurdle treatment against *L. innocua* biofilms; and (3) explore the influence of surface conditions on the practical efficacy of the PAA–steam hurdle inactivation against *L. innocua* biofilms on the selected surfaces.

## 2. Materials and Methods

### 2.1. Activation and Cocktail Preparation of L. innocua Strains

*L. innocua* NRRL B-33197, isolated from an avocado facility, was acquired from the USDA-ARS culture collection of the National Center (NRRL) for Agricultural Utilization Research (Peoria, IL, USA), and *L. innocua* TVS 470 and TVS 471 (isolated from a fresh apple facility) were kindly provided by Dr. Trevor Suslow from the University of California, Davis (Davis, CA, USA). All three strains were stored at −80 °C in Trypticase Soy Broth (Becton, Dickinson, and Company (BD), Sparks, MD, USA) supplemented with 0.6% Yeast Extract (TSBYE, Fisher Scientific, Fair Lawn, NJ, USA) and 20% glycerol. For each experiment, a small aliquot of each frozen culture was transferred in TSBYE and incubated for 24 h at 37 °C. Cells were then sub-cultured in TSBYE followed by another 24 h incubation at 37 °C. The three-strain *L. innocua* cocktail was prepared by mixing an equal volume of each *L. innocua* suspension and centrifuged at 8000× *g* for 15 min at 25 °C, and Modified Welshimer’s Broth (MWB, HiMedia, West Chester, PA, USA) was used to re-suspend cell pellets to have a final level of ~10^8^ CFU/mL.

### 2.2. Preparation of Food-Contact Coupons

SS (AISI 316, number 4 polished finish) was obtained from Washington State University Instrument Shop (Pullman, WA, USA). PET sheets were purchased from Interstate Plastics (Sacramento, CA, USA), and silicone rubber sheets were purchased from Jet Gasket & Seal (Las Vegas, NV, USA). Food-contact surfaces in fresh apple packinghouses may naturally become abraded. To simulate this worn surface condition, SS, PET, and rubber were artificially sanded with 80-grit sandpaper for 5 min as described previously [21]. After preparing the material sheets into 1.5 × 1.5 cm^2^ squares, surface coupons were cleaned following our previous procedure [24]. Briefly, the coupons were soaked in 100% methanol for one hour at room temperature (RT, ~22 °C), then rinsed three times with distilled water. They were then immersed in 70% ethanol for another hour at RT, followed by three additional washes with distilled water. After cleaning, the coupons were dried for 12 h in a hood. Right before *L. innocua* inoculation, the clean coupons were transferred into sterile Petri dishes (VWR International, Radnor, PA, USA) using sterile forceps, with 16 coupons per Petri dish. For sterilization, both the coupons and Petri dishes were exposed to a UV lamp in the biosafety cabinet for 30 min.

For organic soil conditioning, the 16 coupons in each Petri dishes were soaked in 24 mL of 1:10 diluted apple juice for one hour at RT. Apple juice was then removed by vacuum aspiration, and the organic matter-soiled coupons were dried in the biosafety hood for one hour before inoculation [18].

### 2.3. L. innocua Biofilm Formation

To inoculate coupons with and without organic matter, each Petri dish (100 mm) containing 16 coupons was loaded with 24 mL of the above-prepared *L. innocua* cocktail (three-strain, ~10^8^ CFU/mL). All coupons were immersed in *L. innocua* suspension. To ensure uniform inoculation, the suspension was thoroughly vortexed before inoculation, and the Petri dishes were gently shaken after inoculation. The coupons and Petri dishes were then incubated at RT for 168 h to allow *L. innocua* biofilm formation, without changing the growth medium during the incubation period. Coupons remained in the Petri dishes throughout the 7-day incubation period.

### 2.4. Preparation of Sanitizer Solutions

The Bioside^TM^ HS 15% (EnviroTech, Modesto, CA, USA, EPA registration No. 63838-2) was used to prepare the PAA solutions at the levels of 40 and 80 ppm. PAA solutions were prepared freshly with sterile distilled water before each experiment. The concentrations of PAA were verified with a Peracetic Acid Test Kit (AquaPhoenix, Hanover, PA, USA) before each study.

### 2.5. Hurdle Treatment of Saturated Steam and PAA

After 7-day incubation at RT, the biofilm growth medium was removed from the Petri dishes by vacuum aspiration. Surface coupons carrying the 7-day-old *L. innocua* biofilms were washed with sterile phosphate-buffered saline (PBS). During washing, the coupons were immersed in 24 mL of PBS with gentle shaking between each wash, and PBS was removed by vacuum aspiration after each wash; a total of three washes were perfomed. Subsequently, the *L. innocua* biofilms were subjected to PAA sanitization and saturated steam using two different treatment sequences. Details on the steam processing apparatus, steam profile, and surface temperature profiles of SS, PET, and rubber have been previously reported [24].

In the PAA followed by steam treatment sequence, the coupons were transferred into sterile Petri dishes, with one coupon from each surface material placed in the same Petri dish, totaling four Petri dishes. Coupons bearing *L. innocua* biofilms were initially treated with 24 mL of PAA solutions (40 ppm or 80 ppm) for 1 min at RT. Immediately after removing the PAA solution by vacuum aspiration, 24 mL of Dey-Engley Neutralizing Broth (D/E broth, Thermo Scientific™, Oxoid, Waltham, MA, USA) was added into each Petri dish for 5 min at RT. Within 30 min post PAA treatment, the coupons were exposed to saturated steam for 6 s.

In the steam followed by PAA treatment sequence, coupons carrying biofilms were first treated with saturated steam for 6 s. The steamed coupons were then splaced in sterile Petri dishes on ice, with one coupon from each surface material placed in each dish, totaling four Petri dishes. Within 30 min post-steam treatment, the steamed coupons were immersed in 24 mL of PAA solution (40 ppm or 80 ppm) for 1 min at RT, followed by a 5 min neutralization with D/E broth at RT. The PAA solution and D/E neutralization broth were removed by vacuum aspiration.

Following these hurdle treatments, the coupons were placed in 50 mL centrifuge tubes filled with 2 mL of ice-cold PBS and 5~6 glass beads. Controls included 7-day-old biofilms that did not receive any sanitizer or steam treatment. Three independent studies were conducted, with four replicates for each treatment combination within each independent study.

### 2.6. Biofilm Detachment and Enumeration

To collect *L. innocua* cells from each coupon, the 50 mL falcon tubes containing coupons and 2 mL PBS were placed in a sonication bath (SharperTek^®^ ultrasonic, Pontiac, MI, USA) for 5 min (40 kHz, RT), then vortexed for 2 min with a bench-top mixer at maximal speed. Following this, the suspension was 10-fold serially diluted, and 100 μL of each dilution was spread onto duplicated TSAYE plates. For treatments expected to yield low *L. innocua* counts, 1.0 mL of undiluted bacterial suspension was spread onto three TSAYE plates, and the colony counts from these plates were summed. The TSAYE plates were incubated at 37 °C for 2 days before colony enumeration.

### 2.7. Statistical Analysis

Every study was conducted independently three times, with four replicates for each treatment combination and surface type within a single study. Statistical significance between groups was determined using uncorrected Fisher’s LSD with two-way ANOVA (IBM SPSS Statistics, version 19.0 software, Chicago, IL, USA) at a significance level of *p* ≤ 0.05. Data were shown as the average of three independent tests, with results expressed as mean ± standard error mean (SEM).

## 3. Results

### 3.1. Efficacy of Hurdle Treatments

**Figure 1:**
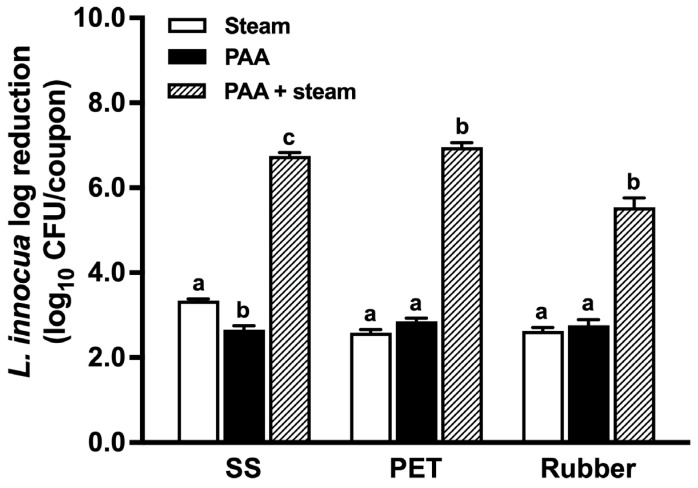
*L. innocua* reductions on food-contact surfaces after different treatments. Seven-day-old *L. innocua* biofilms (6.8–7.0 log_10_ CFU/coupon) on surfaces were treated with peroxyacetic acid (PAA, 80 ppm, 1 min), steam (100 °C, 6 s), or their combination. SS: stainless steel; PET: polyester. Data are presented as mean ± SEM from three independent studies, and each with four replicates. Bars labeled with different letters (a–c) indicate statistically significant differences (*p* < 0.05) for each type of material.

Hurdle treatments combining PAA followed by steam exposure hadhigher efficacy compared to the single treatment of PAA or saturated steam (*p* < 0.05) across all surface materials (Figure 1). PAA at 80 ppm for 1 min, followed by a 6-s steam exposure resulted in 6.8, 7.0, and 5.5 log_10_ CFU/coupon reductions of *L. innocua* biofilms on SS, PET, and rubber, respectively. In contrast, individual treatments with saturated steam (100 °C, 6 s) or 80 ppm PAA for 1 min led to reductions of only 2.6–3.3 or 2.7–2.9 log_10_ CFU/coupon, respectively (Figure 1).

**Figure 2:**
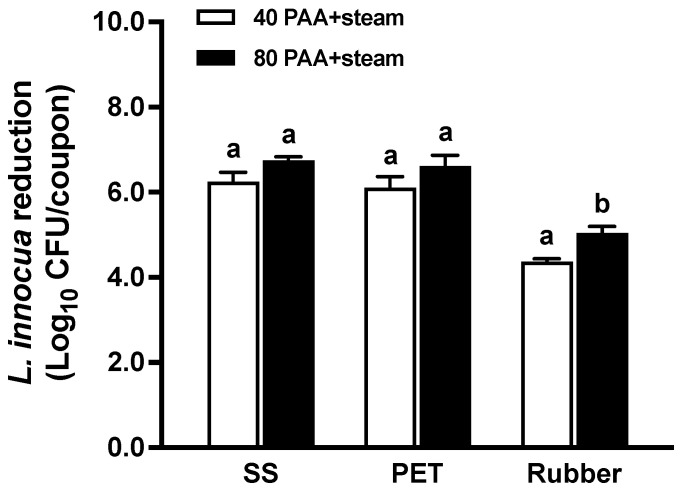
*L. innocua* reductions on food-contact surfaces after hurdle treatments with saturated steam with different concentrations of peroxyacetic acid (PAA). Seven-day-old *L. innocua* biofilms (6.8–7.0 log_10_ CFU/coupon) on surfaces were treated with saturated steam (100 °C, 6 s) in combination with 40 ppm or 80 ppm PAA. SS: stainless steel; PET: polyester. Data are presented as mean ± SEM from three independent studies, each with four replicates per treatment. Bars labeled with different letters (a,b) indicate statistically significant differences (*p* < 0.05).

Reducing PAA concentration from 80 ppm to 40 ppm led to a slight but not statistically significant decrease in efficacy on SS and PET coupons, still resulting in reductions greater than 6 log_10_ CFU/coupon. However, for rubber surfaces, the efficacy of the 40 ppm PAA in combination with steam treatments was significantly lower (*p* < 0.05) than that of 80 ppm PAA with steam treatments, with reductions of 4.4 vs. 5.0 log_10_ CFU/coupon (Figure 2). Across both PAA levels, the hurdle treatments showed comparable efficacy on SS and PET surfaces, but were less effective on rubber surfaces (Figure 2). In addition, the order of PAA and steam treatment did not significantly impact the overall efficacy of the hurdle treatments, regardless of PAA concentrations (Table 1).

### 3.2. Impact of Food Soils on the Efficacy of PAA–Steam Hurdle Treatments

The counts of *L. innocua* in biofilms on clean coupons were 6.8–7.1 log_10_ CFU/coupon, while on soiled coupons, *L. innocua* counts were 7.2–7.3 log_10_ CFU/coupon (Table 2). Organic matter soiling did not significantly affect the efficacy of a quick steam exposure against biofilms on SS, PET, and rubber coupons. However, the presence of food soils significantly reduced the efficacy of hurdle treatments (40 ppm PAA + steam) on SS and PET coupons (*p* < 0.05), with a slight decrease observed on rubber surfaces (Table 2). Overall, a 1 min treatment with 40 ppm PAA followed by a 6 s exposure to saturated steam resulted in 5.6, 5.8, and 4.2 log_10_ CFU/coupon reductions of *L. innocua* on soiled SS, PET, and rubber surfaces, respectively (Table 2).

### 3.3. Influence of Surface Defects on the Efficacies of PAA–Steam Hurdle Treatments

Except for the SS surface, the counts of *L. innocua* in biofilms on worn PET and rubber were higher (*p* < 0.05) compared to those on defect-free surfaces, regardless of the presence of organic matter (Table 3). For SS surfaces, the efficacy of saturated steam alone was reduced (*p* < 0.05) on abraded surfaces compared to new surfaces, with no significant impact observed from organic matter soiling on worn surfaces. Similarly, the effectiveness of PAA at 40 ppm and steam hurdle decreased (*p* < 0.05) on abraded SS, especially when organic matter was present (Table 3).

Despite a greater reduction in *L. innocua* on abraded PET and rubber coupons compared to new surfaces after a 6 s exposure to saturated steam (*p* < 0.05), higher counts of *L. innocua* were recovered from worn PET and rubber surfaces due to their initial higher level (~1 log_10_ CFU/coupon higher) (Table 3). As a result, the efficacy of the 6 s steam treatment was diminished on worn PET and rubber coupons. The presence of apple juice further lowered the effectiveness of the 6 s steam exposure on worn surfaces, with a notable impact on rubber (*p* < 0.05) and a slight effect observed on PET. A similar trend was observed with the 40 ppm PAA and steam hurdle treatment, which displayed reduced efficacy (*p* < 0.05) on abraded PET and rubber coupons in comparison to new surfaces. The presence of food soils further reduced its efficacy (*p* < 0.05).

Overall, surface defects reduced the effectiveness of steam treatment on all tested surfaces, whether combined with 40 ppm PAA or not. Across surface types, the effectiveness of the PAA–steam hurdle was reduced (*p* < 0.05) on worn surfaces conditioned with apple juice. However, the treatment still achieved reductions of 5.1, 5.2, and 4.5 log_10_ CFU/coupon on SS, PET, and rubber coupons under the worst-case conditions (Table 3).

## 4. Discussion

### 4.1. The Effectiveness of PAA–Steam Hurdle against L. innocua Biofilms

PAA is a commonly used disinfectant for surface decontamination in the range of 100–200 ppm without requiring a final rinse [30]. We previously reported that 1 min application of 160 ppm PAA resulted in 3.7–4.3 log_10_ CFU/coupon *L. monocytogenes* reductions on SS, PET, and rubber when treating the 7-day-old biofilms [18]. However, the efficacy of PAA was compromised by organic soiling [17,18] and surface defects [21], underscoring the need to improve its efficacy. In this study, we test the efficacy of PAA in combination with rapid steam exposure against *Listeria* biofilms on major materials used in apple packing lines. The results showed that the combinations of PAA and steam enhanced efficacy (*p* < 0.05) compared to single treatments of PAA or steam alone. For example, PAA at 40 ppm for 1 min followed by a 6 s exposure to steam resulted in an over 6.0 log_10_ CFU/coupon reduction of *L. innocua* biofilms on SS and PET coupons. Similarly, PAA (80 ppm, 1 min) combined with 6 s of steam exposure achieved a 5-log reduction on rubber. The efficacy of this hurdle treatment surpassed that of other related studies. For example, lactic acid (2%, 30 s) used before a 5 s steam exposure caused 4.2 and 3.9 log *L. monocytogenes* reductions on SS and polyvinyl chloride, respectively [23], while hydrogen peroxide (2%, 30 s) combined with a 10 s steam exposure resulted in a 3.9 log *L. monocytogenes* reduction on SS [22]. These differences in the anti-*Listeria* effects of sanitizer–steam hurdles may be due to differences in sanitizer type, concentration, treatment duration, steam processing apparatus, bacterial strains, experiment settings, and other factors. The treatment sequence did not impact the effectiveness of the PAA–steam hurdle, regardless of the PAA concentration or surface type. In alignment with our observation, the order of a non-thermal treatment (e.g., UV-C) and superheated steam hurdle treatment had no impact on collective efficacy against *Bacillus cereus* spores [31].

The anti-*Listeria* efficacy of the PAA–steam hurdle was higher on SS and PET surfaces than on rubber surfaces, regardless of PAA concentrations and surface conditions. In alignment with our finding, lactic acid plus saturated steam treatment exhibited greater efficacy on the SS surface than on polyvinyl chloride in decontaminating 6-day-old *L. monocytogenes* biofilms [23]. Steam also demonstrated greater anti-*Listeria* efficacy on SS than on non-SS surfaces, such as polyvinyl chloride surfaces [25,32]. This difference may be due to variations in the thermal conductivity of different surface materials. For example, the thermal conductivity of SS (13.8 W/mk) was much higher than that of PET (0.18 W/mk) and rubber (0.15 W/mk) [33,34,35], which influenced the surface temperature during saturated steam exposure. Our previous study reported that a 6 s exposure to ~100 °C steam exposure resulted in a surface temperature of ~95 °C on SS, ~93 °C on PET, and ~92 °C on rubber surfaces [24]. Additionally, rubber surfaces are rougher than SS and PET surfaces, with arithmetic mean roughness values of 0.51 µm for rubber, 0.28 µm for SS, and 0.12 µm for PET [21]. Another related study also showed that rubber surfaces had high numbers of micro-cracks, providing numerous niches for *L. monocytogenes* during antimicrobial interventions [36]. Thus, compared to SS and PET materials, the relatively lower surface temperature and higher roughness of rubber surfaces likely increase the resistance of *L. innocua* cells during the PAA and steam hurdle treatments.

### 4.2. The Influence of Surface Conditions on the Efficacy of PAA–Steam Hurdle Treatment

Natural aging due to extended usage and improper surface disinfection methods (e.g., physical brushing) can cause substantial pitting on equipment surfaces, thereby increasing the risk of microbial contamination. *Listeria* contamination was found on various worn food-contact surfaces in multiple food facilities [8,37,38,39]. However, most evaluations of sanitization efficacy have focused on disinfecting *Listeria* biofilms from new, defect-free food-contact surfaces [18,19,25,32,36,40]. Limited information is available on disinfecting surfaces that are not brand new. Schlisselberg and Yaron [41] reported that chlorine treatment was more effective in decontaminating *Salmonella* Typhimurium biofilms on untreated SS compared to mechanically sanded SS surfaces. Similarly, the efficacy of chlorine against *Staphylococcus aureus* biofilms was reduced on scratched polypropylene compared to smooth surfaces [42]. Recently, we reported that anti-*Listeria* efficacy of chemical sanitizers, including PAA, was compromised on worn surface coupons compared to new ones, regardless of surface materials [21]. Herein, we showed that the steam alone or combined with 40 ppm PAA was less effective in decontaminating *Listeria* biofilms on worn surfaces compared to new surfaces. This is consistent with previous findings where saturated steam showed higher anti-*Listeria* efficacy on smooth surfaces, such as watermelon, compared to rougher surface like cantaloupe [43].

This study further demonstrated that organic matter soiling on food-contact surfaces decreased the efficacy of the PAA–steam hurdle in decontaminating *Listeria* biofilms, regardless of surface condition (new or worn) and surface types (SS, PET, and rubber). This finding aligns with our previous studies, where the anti-*Listeria* efficacy of PAA on both new [17,18] and worn surfaces [21] was compromised by apple juice conditioning. Similarly, the decontamination efficacy of PAA and other sanitizers against *L. monocytogenes* biofilms on SS surfaces was reduced by meat residue soiling [44], and chicken serum lowered the bactericidal activity of electrolyzed water against *L. monocytogenes* biofilms [45]. In contrast, our earlier study indicated that the presence of diluted apple juice did not impact the anti-*Listeria* efficacy of saturated steam in decontaminating biofilms from SS, PET, and rubber coupons [24]. Organic matter on surfaces may shield *Listeria* from sanitizer inactivation, either by impeding sanitizers from reaching bacterial cells or by reacting with sanitizer and reducing their efficacy. Despite compromising in effectiveness due to worn surfaces and food soils, our findings suggest that PAA combined with a brief steam intervention, remains an effective strategy against *Listeria* biofilms on food-contact surfaces. Under the worst-case scenario (worn surface soiled with apple juice), a 1 min application of PAA at 40 ppm, followed by a 6 s steam exposure, achieved more than a 5 log_10_ CFU/coupon reduction of *Listeria* biofilms on SS and PET coupons and a ~4.5 log_10_ CFU/coupon reduction on the rubber materials.

## 5. Conclusions

A 1 min treatment of PAA at 40 ppm in combination with a 6 s saturated steam resulted in over a 6 log_10_ CFU/coupon reduction of *L. innocua* biofilms on SS and PET materials, while PAA at 80 ppm, combined with 6 s saturated steam treatment led to ~5 log_10_ CFU/coupon reduction on rubber surfaces. The effectiveness of PAA–steam hurdle treatment was not influenced by the sequence of treatment at the tested PAA concentrations. However, apple juice soiling and/or abraded surfaces reduced the efficacy of the 40 ppm PAA–steam hurdle treatment across all surfaces evaluated. Nonetheless, 1 min application of 40 ppm PAA followed by 6 s saturated steam still achieved ≥ 5 log_10_ CFU/coupon reductions of *L. innocua* on SS and PET coupons under the worst-case scenarios. Although slightly less effective on rubber, with ~4.5 log_10_ CFU/coupon reduction, PAA at 40–80 ppm combined with brief steam treatment presents an effective strategy for surface decontamination.

## Figures and Tables

**Table 1:** Influence of treatment sequence on the efficacy of PAA–steam hurdle treatments on new surfaces.

Surface	PAA Conc. (ppm)	*L. innocua* Reduction in Biofilms (Log_10_ CFU/Coupon)	
		PAA + Steam	Steam + PAA
SS	40	6.25 ± 0.22 ^aA^	6.31 ± 0.25 ^aA^
	80	>6.53 ^aA^	>6.53 ^aA^
PET	40	6.11 ± 0.26 ^aA^	6.27 ± 0.30 ^aA^
	80	6.61 ± 0.26 ^aA^	6.26 ± 0.28 ^aA^
Rubber	40	4.37 ± 0.07 ^aA^	4.61 ± 0.23 ^aA^
	80	5.04 ± 0.16 ^bA^	4.84 ± 0.15 ^aA^

*L. innocua* biofilms (initial level: 6.8–7.0 log_10_ CFU/coupon) on stainless steel (SS), polyester (PET), and rubber were treated with peroxyacetic acid (PAA) for 1 min and 6 s of saturated steam. PAA + steam: PAA treatment followed by saturated steam; steam + PAA: saturated steam treatment followed by PAA. Data are presented as mean ± SEM from three independent studies, each with four replicates. Numbers labeled with different letters (a,b) within each column differ significantly (*p* ≤ 0.05) for the same surface material. Numbers labeled with the same letters (A) within each row do not differ significantly (*p* > 0.05) within each row.

**Table 2:** Influence of food soils on the efficacy of saturated steam with or without 40 ppm PAA.

Surface	Conditions	Initial Levels (Log_10_ CFU/Coupon)	*L. innocua* Reduction in Biofilms (Log_10_ CFU/Coupon)	
			Steam	PAA + Steam
SS	Clean	6.83 ± 0.05	3.34 ± 0.04 ^aA^	6.25 ± 0.22 ^aB^
	Soiled	7.17 ± 0.08	3.56 ± 0.05 ^aA^	5.56 ± 0.18 ^bB^
PET	Clean	7.13 ± 0.09	2.59 ± 0.07 ^aA^	6.11 ± 0.26 ^aB^
	Soiled	7.32 ± 0.06	2.72 ± 0.07 ^aA^	5.76 ± 0.21 ^bB^
Rubber	Clean	7.03 ± 0.09	2.65 ± 0.09 ^aA^	4.37 ± 0.07 ^aB^
	Soiled	7.32 ± 0.08	2.64 ± 0.07 ^aA^	4.17 ± 0.04 ^aB^

*L. innocua* biofilms on stainless steel (SS), polyester (PET), and rubber coupons conditioned with or without diluted apple juice were treated with 40 ppm peroxyacetic acid (PAA) followed by 6 s of steam. Data are presented as mean ± SEM from three independent studies, each with four replicates. Numbers labeled with different letters (a,b) within each column indicate statistically significant difference (*p* ≤ 0.05) for the same surface material. Numbers labeled with different letters (A,B) within each row indicate statistically significant differences (*p* ≤ 0.05) between treatments.

**Table 3:** Impact of surface condition on the efficacy of saturated steam with or without 40 ppm PAA.

Surface	Conditions	Initial Levels (Log_10_ CFU/Coupon)	*L. innocua* Reduction in Biofilms (Log_10_ CFU/Coupon)	
			Steam	PAA + Steam
SS	New, clean	6.83 ± 0.05 ^a^	3.34 ± 0.04 ^aA^	>6.53 ^aB^
	Worn, clean	7.22 ± 0.04 ^a^	2.56 ± 0.04 ^bA^	5.91 ± 0.27 ^bB^
	Worn, soiled	7.15 ± 0.06 ^a^	2.70 ± 0.12 ^bA^	5.08 ± 0.12 ^cB^
PET	New, clean	7.13 ± 0.09 ^a^	2.59 ± 0.07 ^aA^	6.61± 0.26 ^aB^
	Worn, clean	8.28 ± 0.07 ^b^	3.50 ± 0.07 ^bA^	5.69± 0.22 ^bB^
	Worn, soiled	8.18 ± 0.07 ^b^	3.33 ± 0.05 ^bA^	5.18 ± 0.08 ^cB^
Rubber	New, clean	7.03 ± 0.09 ^a^	2.65 ± 0.09 ^aA^	4.37 ± 0.07 ^aB^
	Worn, clean	8.00 ± 0.05 ^b^	3.23 ± 0.10 ^bA^	4.84 ± 0.04 ^bB^
	Worn, soiled	7.97 ± 0.07 ^b^	2.79 ± 0.13 ^aA^	4.49 ± 0.04 ^cB^

Seven-day-old *L. innocua* biofilms on worn stainless steel (SS), polyester (PET), and rubber surface, conditioned with or without apple juice, were treated with steam (100 °C, 6 s) with or without 40 ppm peroxyacetic acid (PAA). Biofilms on new, clean surfaces served as control. Data are presented as mean ± SEM from three independent studies, each with four replicates. Numbers labeled with different letters (a–c) within each column differ significantly (*p* < 0.05) for the same surface material. Numbers labeled with different letters (A,B) within each row differ significantly (*p* < 0.05).

## Data Availability

The original contributions presented in the study are included in the article, further inquiries can be directed to the corresponding author.
